# Knowledge, attitudes and practices about research misconduct among medical residents in southwest China: a cross-sectional study

**DOI:** 10.1186/s12909-024-05277-6

**Published:** 2024-03-14

**Authors:** Lulin Chen, Yizhao Li, Jie Wang, Yue Li, Xiaoli Tan, Xiaoyan Guo

**Affiliations:** 1 Department of Preventive Health, The Second Nanning People’s Hospital, The Third Affiliated Hospital of Guangxi Medical University, Nanning, People’s Republic of China; 2 Department of Science and Education, The Second Nanning People’s Hospital, The Third Affiliated Hospital of Guangxi Medical University, Nanning, People’s Republic of China

**Keywords:** Knowledge, Attitude, Practice, Research misconduct, Residents, Retraction

## Abstract

**Background:**

With the emergence of numerous scientific outputs, growing attention is paid to research misconduct. This study aimed to investigate knowledge, attitudes and practices about research misconduct among medical residents in southwest China.

**Methods:**

A cross-sectional study was conducted in southwest China from November 2022 through March 2023. The links to the questionnaire were sent to the directors of the teaching management department in 17 tertiary hospitals. Answers were collected and analyzed. Logistic regression analysis was performed to explore the factors associated with research misconduct among residents.

**Results:**

6200 residents were enrolled in the study, and 88.5% of participants attended a course on research integrity, but 53.7% of participants admitted to having committed at least one form of research misconduct. Having a postgraduate or above, publishing papers as the first author or corresponding author, attending a course on research integrity, lower self-reported knowledge on research integrity and lower perceived consequences for research misconduct were positively correlated to research misconduct. Serving as a primary investigator for a research project was negatively associated with research misconduct. Most residents (66.3%) agreed that the reason for research misconduct is that researchers lack research ability.

**Conclusions:**

The high self-reported rate of research misconduct among residents in southwest China underscores a universal necessity for enhancing research integrity courses in residency programs. The ineffectiveness of current training in China suggests a possible global need for reevaluating and improving educational approaches to foster research integrity. Addressing these challenges is imperative not only for the credibility of medical research and patient care in China but also for maintaining the highest ethical standards in medical education worldwide. Policymakers, educators, and healthcare leaders on a global scale should collaborate to establish comprehensive strategies that ensure the responsible conduct of research, ultimately safeguarding the integrity of medical advancements and promoting trust in scientific endeavors across borders.

## Introduction

With the emergence of numerous scientific outputs, growing attention is being paid to research misconduct throughout the world [1]. Chinese scientific output has increased dramatically in recent years, and accounts for 23.4% of the total scientific papers and 27.2% of the top 1% most frequently cited papers between 2018 and 2020, overtaking the US [2]. The overwhelming amount of scientific outputs have also brought international attention to research misconduct in China. According to the Retraction Watch Database, 5561 articles were retracted from China in 2023, accounting for 78.5% of total retracted articles around the world [3]. A research report from the Nature indicates that more than 17,000 retractions with Chinese co-authors have been produced in China’s first nationwide review of retractions and scientific misconduct since 2021 [4]. An investigation on scientific misconduct in Chinese tertiary hospitals suggested that approximately 40% of researchers admitted to having committed research misconduct, with inappropriate authorship being the most common form [5]. A survey on nursing students reported that 44.1% of participants were involved in at least one form of research misconduct [6]. Research misconduct leads to a variety of detrimental consequences, such as misleading other researchers and hindering scientific innovation and development [7]. The medical science field should impose stricter requirements for research integrity due to its involvement in health status [8].

Residents’ participation in research can encourage academic careers, enhance clinical reasoning, promote evidence-based practice, and ultimately improve patient outcomes [9, 10]. In some countries, residency programs mandatorily teach the basic principles of research alongside other scholarly activities [11]. Earlier studies with different results have been conducted to evaluate residents’ knowledge, attitudes and practices toward research. Most residents regarded research activity as an important part of their career, but a lack of protected time and experience in research skills and overload of resident clinical work were the major barriers for research, and insufficient research training, limited access to research methodologies, and peer pressure also had a negative impact [1, 12]. These barriers may lead to research misconduct. 67.4% of the researchers held the idea that a lack of research ability was the reason for research misconduct [13]. Therefore, residents are likely to conduct research misconduct due to these barriers.

Although many studies have been conducted to assess the researchers’ knowledge, attitudes, and practices toward research [12, 14, 15], there is still limited knowledge about the prevalence and associated factors of research misconduct among residents around the world. Therefore, we conducted this cross-sectional study to identify these issues in southwest China, and we also investigated the perceived reasons for research misconduct among residents.

## Materials and methods

### Study design

A cross-sectional investigation was conducted in southwest China from November 2022 through March 2023. Participants were residents at 17 tertiary hospitals from 8 cities, including Nanning, Liuzhou, Yulin, Qinzhou, Wuzhou, Beihai, Guilin, and Baise. Of the 17 hospitals, 9 were provincial, and 8 were municipal. We applied convenience sampling in the study. The links to the questionnaire were sent to the directors of the teaching management department in the above hospitals, who were asked to forward the investigation to their residents. The informed consent form and questionnaire were both completed online. After reading and submitting the informed consent form, residents could choose to fill out the questionnaire, and then the questionnaire was returned anonymously without available information to identify residents.

### Questionnaire design

The questionnaire was designed according to previous studies. It consists of 5 parts, including demographic characteristics and research experience, knowledge of research integrity, perceived consequences for research misconduct, residents’ involvement in research misconduct and perceived reasons for research misconduct. All the questions were closed-ended. The responses to parts 2–3 were expressed by a 5-point Likert-type scale.

#### Knowledge about research integrity

We designed 12 questions according to an official document released by the Ministry of Education of the People’s Republic of China [16] and a previous study [5]. Of the 12 questions, 9 were used to evaluate research integrity during the process of writing research proposals or applying for research projects, conducting research, and publishing papers, and the other 3 were about authorship, research ethics and documentation on scientific integrity issued by regulatory authorities, respectively. The questions could be answered on a scale from 1 to 5 (completely don’t know = 1, know a little = 2, know some = 3, know = 4, completely know = 5). The total scores range from 12 to 60, with higher scores indicating a higher level of knowledge about research integrity. The Cronbach’s α of these items was 0.980, and the KMO index was 0.963.

#### Perceived consequences for research misconduct

Residents’ perceived consequences for research misconduct were assessed using a 7-item checklist with the reference to a previous study [17]. The questions could be answered on a scale from 1 to 5 (no influence = 1, a little influence = 2, moderate influence = 3, strong influence = 4, very strong influence = 5). The total scores range from 7 to 35. Higher scores indicate a greater severity of perceived consequences for research misconduct. The Cronbach’s α of these items was 0.972, and the KMO index was 0.940.

#### Residents’ involvement in research misconduct

This part was designed according to the definition of research misconduct by The Ministry of Education of the People’s Republic of China, including 5 common situations [16]. In addition, multiple submissions and duplicate publications were also added. The frequencies of research misconduct were divided into 5 levels, including never, 1 time, 2 times, 3 times and ≥ 4 times.

#### Perceived reasons for research misconduct

We evaluated residents’ perceived reasons for research misconduct using a 7-item checklist based on a previous study [17]. The checklist consists of both internal and external reasons, such as researchers lack research ability, and researchers are influenced by academic environment. The questions could be answered with “agree”, “neutral” or “disagree”.

### Data analysis

SPSS 25.0 (IBM, Chicago, IL, USA) was used to analyze the data. Categorical variables are described using frequencies and percentages, and continuous variables are expressed as the mean (M) and standard deviation (SD). In our study, the 5-point Likert scale was summed and a total score was obtained. The mean score was calculated, and those who scored at or above the mean were identified as the high group, while others were classified as the low group. Logistic regression analysis was performed to explore the factors associated with research misconduct among residents. A *P value* less than 0.05 was considered statistically significant.

## Results

A total of 6553 questionnaires were collected, of which 6200 were valid after excluding those with contradictory records, with an effective rate of 94.61%. Residents’ demographic characteristics are shown in Table 1. 60.2% of the residents had an undergraduate degree or below, and 39.8% had a postgraduate degree or above. In terms of research and publishing experience, 37.6% of the participants served as a primary investigator for a research project and 38.2% published a paper as the first author or corresponding author. Most residents (88.5%) attended a course on research integrity.

**Table 1:** Characteristics of participants (*n* = 6200)

Characteristics	n(%)
Residency year	
1	1535(24.8)
2	4147(66.9)
3	518(8.4)
Educational status	
Undergraduate or below	3734(60.2)
Postgraduate or above	2466(39.8)
Serving as a primary investigator for a research project	
No	3871(62.4)
Yes	2329(37.6)
Publishing papers as the first author or corresponding author	
No	3830(61.8)
Yes	2370(38.2)
Attending a course on research integrity	
No	714(11.5)
Yes	5486(88.5)

Table 2 shows residents’ self-reported knowledge on research integrity. The highest scores were research ethics and disposition of research misconduct, and the lowest score was documentation on scientific integrity issued by regulatory authorities. The average total score of self-reported knowledge among residents was 39.44 ± 14.46.

**Table 2:** Self-reported knowledge of residents regarding research integrity (*n* = 6200)

Items	n(%)					M ± SD
	Completely know	Know	Know some	Know a little	Completely don’t know	
Documentation on scientific integrity issued by regulatory authorities	1205(19.4)	1869(30.1)	891(14.4)	1104(17.8)	1131(18.2)	3.14 ± 1.40
Definition of research Integrity	1243(20.0)	2194(35.4)	900(14.5)	1091(17.6)	772(12.5)	3.33 ± 1.31
Definition of fabrication	1117(18.0)	1971(31.8)	1008(16.3)	1144(18.5)	960(15.5)	3.29 ± 1.36
Definition of falsification	1338(21.6)	1985(32.0)	963(15.5)	994(16.0)	920(14.8)	3.18 ± 1.35
Definition of plagiarism	1335(21.5)	1964(31.7)	945(15.2)	1003(16.2)	953(15.4)	3.28 ± 1.37
Inappropriate authorship	1127(18.2)	2147(34.6)	1267(20.4)	990(16.0)	669(10.8)	3.33 ± 1.25
Concept of multiple submission	1119(18.0)	2299(37.1)	1009(16.3)	939(15.1)	834(13.5)	3.31 ± 1.30
Concept of duplicate publication	1352(21.8)	2115(34.1)	855(13.8)	1015(16.4)	863(13.9)	3.34 ± 1.35
Citation rules	1161(18.7)	2016(32.5)	1126(18.2)	913(14.7)	984(15.9)	3.24 ± 1.34
Research ethics	1338(21.6)	2065(33.3)	1083(17.5)	958(15.5)	756(12.2)	3.37 ± 1.31
Use of Research Funding	1088(17.5)	2253(36.3)	911(14.7)	1039(16.8)	909(14.7)	3.25 ± 1.33
Disposition of research misconduct	1418(22.9)	2005(32.3)	971(15.7)	1058(17.1)	748(12.1)	3.37 ± 1.33
Total score	-	-	-	-	-	39.44 ± 14.46

As shown in Table 3, of the 7 listed consequences, the entire academic environment was the most perceived consequence with a score of 3.68 ± 1.29 and personal academic reputation was the least perceived consequence with a score of 3.46 ± 1.37. The average total score of perceived consequences for research misconduct among residents was 25.16 ± 8.47.

**Table 3:** Perceived consequences for research misconduct among residents (*n* = 6200)

Variables	n(%)					M ± SD
	Very strong influence	Strong influence	Moderate influence	A little influence	No influence	
Personal academic reputation	1607(25.9)	2219(35.8)	720(11.6)	756(12.2)	898(14.5)	3.46 ± 1.37
The reputation of the institution and academic community	1846(29.8)	2249(36.3)	780(12.6)	818(13.2)	507(8.2)	3.66 ± 1.25
The normal progression of research activities	1783(28.8)	2292(37.0)	749(12.1)	647(10.4)	729(11.8)	3.61 ± 1.32
The rational allocation of research resources	1684(27.2)	2435(39.3)	718(11.6)	746(12.0)	616(9.9)	3.62 ± 1.28
The entire academic environment	2034(32.8)	2016(32.5)	862(13.9)	714(11.5)	574(9.3)	3.68 ± 1.29
Public trust in researchers	1714(27.6)	2190(35.3)	762(12.3)	718(11.6)	816(13.2)	3.53 ± 1.35
Research integrity throughout the society	1752(28.3)	2284(36.8)	770(12.4)	752(12.1)	642(10.4)	3.61 ± 1.29
Total score	-	-	-	-	-	25.16 ± 8.47

Table 4 presents the residents’ involvement in research misconduct. 3331 residents (53.7%) admitted to having committed at least one of the seven listed forms of research misconduct (Table 5). The most common type of research misconduct was multiple submissions (50.6%), and the least common type was buying and selling papers, letting other people write papers, or writing papers for others (46.7%).

**Table 4:** Residents’ involvement in research misconduct (*n* = 6200)

Variables	Never(%)	1 time(%)	2 times(%)	3 times(%)	≥ 4 times(%)	Self-reported rate
Multiple submissions	3063(49.4)	585(9.4)	947(15.3)	846(13.6)	759(12.2)	3137(50.6)
Duplicate publication	3302(53.3)	679(11.0)	761(12.3)	849(13.7)	609(9.8)	2898(46.7)
Buying and selling papers, letting other people write papers, or writing papers for others	3303(53.3)	577(9.3)	811(13.1)	948(15.3)	561(9.0)	2897(46.7)
Plagiarizing or misappropriating others’ academic achievements	3179(51.3)	662(10.7)	990(16.0)	815(13.1)	554(8.9)	3021(48.7)
Falsifying research data, materials, literature or annotations, or fabricating research results	3162(51.0)	592(9.5)	816(16.2)	960(15.5)	670(10.8)	3038(49.0)
Providing false academic information in the process of the project application, application of achievement, award, or title, or degree application	3183(51.3)	689(11.1)	934(15.1)	790(12.7)	604(9.7)	3017(48.7)
Joining the authorship without participating in the research or creation, listing others as authors without their permission, listing fake names as authors, or not listing authors who made contributions to the research and manuscript	3250(52.4)	526(8.5)	641(10.3)	1083(17.5)	700(11.3)	2950(47.6)

**Table 5:** Logistic regression analysis of research misconduct (*n* = 6200)

Characteristics	Research misconduct		OR(95%CI)	P-value
	No (%) *n* = 2869	Yes (%) *n* = 3331		
Residency year				
1	593(20.7)	942(28.3)	1.000	-
2	1910(66.6)	2237(67.2)	1.137(0.954–1.354)	0.151
3	366(12.8)	152(4.6)	0.809(0.712–1.126)	0.192
Educational status				
Undergraduate or below	2233(77.8)	1501(45.1)	1.000	-
Postgraduate or above	636(22.2)	1830(54.9)	2.457(2.076–2.909)	< 0.01
Serving as a primary investigator for a research project				
No	1434(50.0)	2437(73.2)	1.000	-
Yes	1435(50.0)	894(26.8)	0.600(0.510–0.715)	< 0.01
Publishing papers as the first author or corresponding author				
No	2184(76.1)	1646(49.4)	1.000	-
Yes	685(23.9)	1685(50.6)	4.271(3.641–5.009)	< 0.01
Attending a course on research integrity				
No	495(17.3)	219(6.6)	1.000	-
Yes	2374(82.7)	3112(93.4)	4.242(3.226–5.579)	< 0.01
Grouped by self-reported knowledge regarding research integrity				
High	2163(75.4)	1250(37.5)	1.000	-
Low	706(24.6)	2081(62.5)	2.374(1.937–2.908)	< 0.01
Grouped by perceived consequences for research misconduct				
High	2707(94.4)	1162(34.9)	1.000	-
Low	162(5.6)	2169(65.1)	20.411(16.325–25.52)	< 0.01

We involved all the demographic characteristics, self-reported research integrity knowledge and perceived consequences for research misconduct to determine the factors associated with research misconduct, and the results are displayed in Table 5. Having a postgraduate degree or above (OR = 2.457, 95% CI = 2.076–2.909, *P* < 0.01), publishing papers as the first author or corresponding author (OR = 4.271, 95% CI = 3.641–5.009, *P* < 0.01), attending a course on research integrity (OR = 4.242, 95% CI = 3.226–5.579, *P* < 0.01), lower self-reported knowledge on research integrity (OR = 2.374, 95% CI = 1.937–2.908, *P* < 0.01) and lower perceived consequences for research misconduct (OR = 20.411, 95% CI = 16.325–25.528, *P* < 0.01) were positively correlated to research misconduct, and serving as a primary investigator for a research project (OR = 0.600, 95% CI = 0.510–0.715, *P* < 0.01) was negatively associated with research misconduct.

Table 6 depicts the perceived reasons for research misconduct among residents. The most perceived reason for research misconduct was that researchers lack research ability (66.3%), and the reason that researchers are influenced by academic environment ranked the second (65.7%). The reason with the lowest agreement rate was that there is a lack of research integrity training (62.0%).

**Table 6:** Perceived reasons for research misconduct among residents (*n* = 6200)

Variables	Agree(%)	Neutral(%)	Disagree(%)
Researchers do not understand the content of research integrity	3852(62.1)	766(12.4)	1582(25.5)
Researchers lack research ability	4113(66.3)	825(13.3)	1262(20.3)
Researchers deviate in personal value and lack of academic ethics	3996(64.4)	856(13.8)	1348(21.8)
There is a lack of research integrity training	3846(62.0)	1012(16.3)	1342(21.7)
Researchers are influenced by academic environment	4070(65.7)	647(10.4)	1483(23.9)
There is a lack of academic supervision	3994(64.4)	917(14.8)	1489(20.8)
There exist defects of academic quantitative evaluation	3903(62.9)	890(14.4)	1407(22.7)

## Discussion

To our knowledge, this study is the first survey on residents’ knowledge, attitudes and practices towards research misconduct and factors associated with research misconduct in China. The questionnaire applied has good reliability and validity referencing previous studies. The results may help policy-makers and hospital managers identify key residents who tended to conduct research misconduct and design the content of residency training. Our study suggests that a limited number of residents served as a primary investigator for a research project (37.6%) and published a paper as the first author or corresponding author (38.2%). Additionally, 53.7% admitted to having committed research misconduct. The average total score of self-reported knowledge among residents was 39.44 ± 14.46 (ranging from 12 to 60). The average total score of perceived consequences for research misconduct among residents was 25.16 ± 8.47 (ranging from 7 to 35). Previous studies have already shown the worrisome prevalence of self-reported research misconduct among medical faculty members [5, 12, 18]. Our study revealed worse results among residents in hospitals, which demonstrated the insufficiency of research integrity management in hospitals.

In our study, multiple submissions (50.6%) was the most frequent form of research misconduct, and 12.2% of the residents conducted multiple submissions ≥ 4 times. Since multiple submissions was only perceived as a severe deviance by scientific journals, some researchers regarded it as little apparent harm [19]. The Ministry of Education of the People’s Republic of China has not emphasized the severity of multiple submissions [16], and it was not even considered as research misconduct by some researchers [20]. Falsifying research data, materials, literature or annotations, or fabricating research results was the second most common form of research misconduct with an alarming self-reported rate of 49.0%. Bjørn et al. reported that 10.0% of participants believed the common incidence of falsification, fabrication, and plagiarism (FFP) and some respondents were willing to conduct FFPs based on their perceived true conclusions [21]. When researchers applied for a grant, FFPs were more acceptable and regarded as not important as in publications, so the actual prevalence of FFPs may be worse than expected.

Residents with a postgraduate degree or above may be more likely to conduct research misconduct. Oren et al. [22] also reported that PhD nurses tend to fabricate, select or omit data to improve their chances of publication. Majid et al. [23] suggested that postgraduate students have a higher estimation of research misconduct than undergraduate students. The additional statistical skills in postgraduate students may make it easier for them to fabricate, select or omit data, and the desire to be successful drives them to conduct research misconduct [19]. Besides, the contradiction between the limited time and overloaded work, and high demand for postgraduates’ scientific achievement may lead to the incidence of research misconduct. Those serving as a primary investigator for a research project have a lower inclination to conduct research misconduct. This may be related to the experience of researchers. Primary investigators usually have relatively rich research experience and knowledge, whereas junior researchers usually have poor knowledge of research misconduct [20]. On the other hand, the primary investigator bears the greatest responsibility, which makes them pay more attention to research integrity, and have more opportunities to be exposed to relevant knowledge and cases of research integrity.

In our study, most residents (88.5%) attended a course on research integrity, which was surprisingly contrary to the high prevalence of research misconduct. Attending a course on research integrity contributed to research misconduct in the study, indicating the shortcomings in the current research integrity courses and urgency to update and implement the content of courses. Traditional lectures are still the most commonly used teaching method in China, and are significantly less effective and efficient than the seminar teaching method or the combined problem-based learning and case-based learning teaching method [24, 25]. Furthermore, there is no research integrity course specifically designed for residents, and they are usually trained together with hospital staff. The academic environment is complex and junior residents usually learn about research integrity from their supervisors or senior students [20]. This may influence their knowledge, attitudes and practices towards research misconduct. Further studies should be conducted to explore resident’ perceptions of research integrity courses, and in-depth interview method should be adopted among residents to optimize course design.

Lower self-reported knowledge was associated with higher research misconduct prevalence. We also collected the self-reported reasons for research misconduct from residents. The top 3 reasons are “researchers lack research ability”, “researchers are influenced by academic environment” and “researchers deviate in personal value”. These results indicate that a lack of personal research ability and knowledge about research misconduct leads to residents’ involvement in research misconduct. The reputation and income of medical staff are closely associated with the professional title, and research achievements play an important role in the professional title promotion system and the evaluation system in Chinese tertiary hospitals, such as publication of papers or grant application. The contradiction between research ability and promotion pressure would contribute to the incidence of research misconduct. Previous studies also suggest that promotion pressure and individual morality are the main perceived reasons for research misconduct [1, 5]. Consistent with earlier surveys, personal morality was the main influencing factor, suggesting it is important to enhance personal morality [19]. Lower perceived consequences for research misconduct were significantly correlated to research misconduct with the enormous OR, and this may provide clues for the design of training. Few researchers considered that education on research misconduct has an effect on reducing the incidence of research misconduct [5]. Therefore, more courses focusing on consequences for research misconduct should be conducted for residents to reduce the incidence of research misconduct.

Our results reflect the weakness of research integrity courses, the importance of perceived consequences and practices for research misconduct, and the factors linked with research misconduct among residents. Those who were postgraduate or above, had lower scores of research misconduct knowledge and perceived consequences, did not serve as a primary investigator for a research project, published papers as the first author or corresponding author, and attended a course on research integrity tended to conduct research misconduct. This revealed a troubling phenomenon in which residents with research experience almost have a tendency to conduct research misconduct despite being trained, suggesting the necessity for the reformation of residents’ education. More attention should be paid to residents’ education by hospital managers and policy-makers, and we propose several recommendations to improve research integrity. First, government administrations should emphasize the importance of research integrity, and include research integrity in compulsory courses by residency training programs, and the seminar teaching method or the combined problem-based learning and case-based learning teaching method could be applied to improve the effectiveness of courses, instead of relying on traditional lectures. Second, in-depth interview with residents may be conducted to optimize curriculum design, and consequences for research misconduct should be emphasized in the courses, and multiple submissions and duplicate publication need to be highlighted due to their high prevalence. Third, an auditing and surveillance system can be implemented in hospitals, and the department in charge of research integrity should be set up and maintain its authority and independence, and the in-hospital review process should be strictly conducted before residents’ scholar activity.

The study has a few limitations. First, convenience sampling was applied due to the sensitivity of research misconduct, which may affect the results. Furthermore, the questionnaire was derived from self-report, and bias may be involved in the process despite assurances of anonymity. Finally, although based on other studies, the questionnaire was self-designed and measurements may differ from the study objectives.

## Conclusions

The high self-reported rate of research misconduct among residents in southwest China underscores a universal necessity for enhancing research integrity courses in residency programs. The ineffectiveness of current training in China suggests a possible global need for reevaluating and improving educational approaches to foster research integrity. Addressing these challenges is imperative not only for the credibility of medical research and patient care in China but also for maintaining the highest ethical standards in medical education worldwide. Policymakers, educators, and healthcare leaders on a global scale should collaborate to establish comprehensive strategies that ensure the responsible conduct of research, ultimately safeguarding the integrity of medical advancements and promoting trust in scientific endeavors across borders.

## Data Availability

The data used during the study are available from the corresponding author on reasonable requests.
